# 

**Published:** 1895-06-29

**Authors:** 


CONTENTS.

The Hospital Problem in London - 213

The Storage of Potable Water - 214

Modern Medico-Psychology and Psychiatry. By T. S. Clouston, M.D. - 215

Medical Progress and Hospital Clinics—

Notes on the Effect of Injuries to different parts of the Cranium - 219

Progress in Medicine.—Infective Diseases - 220

Progress in Surgery.—Fractures and Dislocations - 223

New Appliances and Things Medical - 224

Social Problems: Medicine and Missionaries - 216

Annotations.—The Doctor's Twopence—Egomania—A Good Example - 217

Notes and News - 218

The Institutional Workshop—

Within the Hospitals.—Westminster Hospital - 225

Construction Notes - 226

Scraps and Gleanings - 226

The Metropolitan Hospital Sunday Fund - 227

EXTRA SUPPLEMENT.—THE NURSING MIRROR.

News from the Nursing World.—The Nightingale Training School—Preliminary Training—"The Hospital" Convalescent Fund—An Unforeseen Conclusion—Slighted Recommendations—Provision for Incurable Children—Nurses in Cheltenham—Nursing by Sisterhoods—Training in Paris—Short Items - lxxxiii

Elementary Anatomy and Surgery for Nurses. By W. McAdam Eccles, M.D., M.S., F.R.C.S. XXIII.—The Excretory System—The Absorptive System - lxxxv

Royal National Pension Fund - lxxxv

Nursing in Ireland - lxxxvi

Royal British Nurses' Association - lxxxvi

Appointments - lxxxv.

A Paris School of Massage - lxxxvi

Nurses at Bradford Infirmary - lxxxvi

Lady Dufferin's Fund - lxxxvii

Holidays and Health - lxxxviii

Nursing in the U.S.A. - lxxxviii

Nurses on the Labrador - lxxxviii

Notes from Germany - lxxxix

Notes and Queries - lxxxix

Everybody's Opinion - xc

Novelties for Nurses - xc

A Handsome Legacy - xc

